# Uncertainty Quantification of a Multiscale Model for In-Stent Restenosis

**DOI:** 10.1007/s13239-018-00372-4

**Published:** 2018-08-22

**Authors:** Anna Nikishova, Lourens Veen, Pavel Zun, Alfons G. Hoekstra

**Affiliations:** 1 Computational Science Lab, Institute for Informatics, Faculty of Science, University of Amsterdam, Amsterdam, The Netherlands; 2 Netherlands eScience Center, Amsterdam, The Netherlands; 3 ITMO University, Saint Petersburg, Russia

**Keywords:** In-Stent Restenosis model, Multiscale simulation, Uncertainty quantification, Sensitivity analysis

## Abstract

**Purpose:**

Coronary artery stenosis, or abnormal narrowing, is a widespread and potentially fatal cardiac disease. After treatment by balloon angioplasty and stenting, restenosis may occur inside the stent due to excessive neointima formation. Simulations of in-stent restenosis can provide new insight into this process. However, uncertainties due to variability in patient-specific parameters must be taken into account.

**Methods:**

We performed an uncertainty quantification (UQ) study on a complex two-dimensional in-stent restenosis model. We used a quasi-Monte Carlo method for UQ of the neointimal area, and the Sobol sensitivity analysis (SA) to estimate the proportions of aleatory and epistemic uncertainties and to determine the most important input parameters.

**Results:**

We observe approximately 30% uncertainty in the mean neointimal area as simulated by the model. Depending on whether a fast initial endothelium recovery occurs, the proportion of the model variance due to natural variability ranges from 15 to 35%. The endothelium regeneration time is identified as the most influential model parameter.

**Conclusion:**

The model output contains a moderate quantity of uncertainty, and the model precision can be increased by obtaining a more certain value on the endothelium regeneration time. We conclude that the quasi-Monte Carlo UQ and the Sobol SA are reliable methods for estimating uncertainties in the response of complicated multiscale cardiovascular models.

## Introduction

Cardiac diseases are a leading cause of mortality in developed countries.^16^ Coronary artery stenosis, or abnormal narrowing, is a particularly widespread and potentially fatal cardiac disease. This narrowing is often corrected by stenting the affected artery. There are multiple types of stents currently in use, ranging from simple bare metal stents, to more advanced stents, for example ones eluting growth-inhibiting drugs, ones designed to capture endothelial progenitor cells, and to bioresorbable polymer scaffolds.^10^

In-stent restenosis (ISR) is the process of excessive neointima formation in an artery following balloon angioplasty and stenting, leading to a renewed narrowing of the artery. In 5% to 10% cases it requires a repeat revascularization of the target lesion.^7^ Restenosis is caused by damage to the vessel wall and by disturbed flow patterns in the stented segment.^10^ Restenosis is an important complication of the stenting procedure, and because of that, it has been studied extensively in clinical trials, reviewed e.g., in Ref. 8, *in-vivo* animal experiments, reviewed e.g., in Ref. 9, and also by using computational models.^3^,^5^,^13^,^18^,^34^ The computational models of ISR usually represent cells by agents, either freely moving^5^,^18^,^34^ or placed on a lattice.^3^,^13^ These agents take cues from the blood flow, concentration of drugs eluted from the stent, and from the vessel damage and mechanics, which affect the growth and proliferation of the cells.

We perform an Uncertainty Quantification (UQ) of an off-lattice in-stent restenosis model previously developed by Tahir *et al*.,^30^ where we measure the precision of the model response, not the accuracy. The model is subject to both aleatory and epistemic uncertainty. It includes random variables which represent the stochastic nature of the system. It also includes uncertain parameters, which theoretically can be known precisely, although this is a rare case in practice. Here, we study our two-dimensional version of the ISR model.^5^,^29^,^30^ We do this to provide a proof-of-concept, using a model that is computationally relatively cheap, in preparation for a more in-depth study of our later, more physiological and more computationally expensive three-dimensional ISR model.^34^ Our ultimate goal was to access the model result sensitivity to the uncertainties in the inputs and stochastic parameters, in order to be aware of the result uncertainty and the inputs which cause a greater effect on it. This will allow us to be aware of the result uncertainty quantities, when model results are analyzed, and of the inputs, which value should be as precise as possible.

A model description is given in Sect. 2. Next, in Sect. 3, we give a brief overview of the UQ method we used for measuring uncertainties, as well as of the Sobol method for the analysis of the effect of uncertain inputs on the model result. In Sect. 4, we present the results, and we finish with a discussion and conclusions in Sect. 5.

## The ISR Model

The ISR2D model is a two-dimensional representation of the ISR process.^6^,^30^ Its domain is a longitudinal section of the artery, in which five subprocesses take place: stent deployment, post-deployment smooth muscle cell proliferation into the lumen, blood flow through the lumen, re-endothelialization, and diffusion of antiproliferative drugs from the stent into the tissue. As these processes take place at different temporal scales, ISR2D is a multiscale model.^4^–^6^

**Figure 1:**
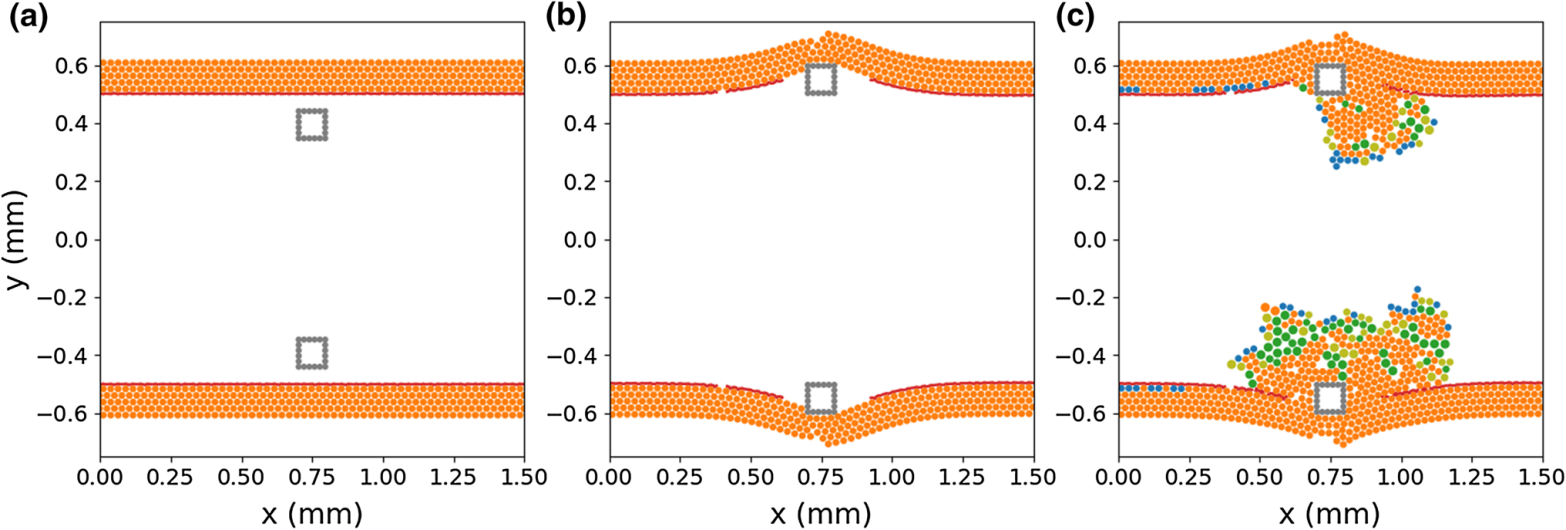
ISR2D initial state, post-deployment state, and reendothelialization pattern. Orange circles are individual SMCs, red circles are IEL agents, and grey circles make up the stent struts. Green cells are synthetic and proliferating, blue are nitric oxide (NO) inhibited. Note the rupture of the IEL in the vicinity of the strut, and subsequent SMC proliferation in this area.

Initially, the simulated artery consists of lower and upper arterial walls (see Fig. 1a). These comprise a tunica media, consisting of multiple layers of smooth muscle cells (SMCs), and a layer of Internal Elastic Lamina (IEL) elements,^30^ all represented by freely moving agents. The adventitia is not modelled explicitly. The initial thickness of the tunica intima is assumed to be negligible, and the endothelial cells are not modelled explicitly. However, an implicit representation of the regenerated endothelium as an attribute of the SMCs (see below) is an important part of the model.^31^ As the only function of the endothelium in this model is to inhibit SMC growth, the lack of endothelium cover on the IEL agents does not affect the results. Square stent struts, also consisting of freely moving agents, are located within the lumen, at some distance from the wall.

The stent deployment process gradually moves the struts outwards to the desired deployment depth, deforming the wall through force-based interaction between the agents. Touching agents are subject to an adhesive Hertzian contact force, while non-touching but nearby agents are attracted by a Hookean force representing the extracellular matrix. Cells at the left and right edges of the domain are constrained horizontally as a boundary condition. The strut agents are fixed in place at a stepwise increasing distance, and forces are equilibrated, forcing the wall to conform. If an agent sustains excessive mechanical stress, or also strain for IEL agents, it is removed. The endothelium is assumed to be completely removed by the angioplasty and stent deployment procedure. While we have not validated this 2D model against known mechanical properties of the arterial wall, our 3D version of the cell interaction model has been validated, see Ref. 14.

Figure 1b shows the resulting initial conditions for the second phase of the simulation. Stent deployment has deformed the arterial wall, and IELs have been removed due to excessive hoop strain (near the strut) as well as longitudinal strain (around *x* = 0.4 mm), exposing the SMCs to the blood flow. No SMCs are covered by functional endothelium at this point.

During the second phase, the exposed SMCs have changed from contractile to synthetic, and traverse the cell cycle, proliferating into the lumen. The neointima is modelled as consisting mainly of SMCs. At each step of the model, each endothelium-free SMC becomes covered by functional endothelium with a time-varying probability calculated to achieve a certain coverage scenario.^29^ Figure 1c shows the model state after some growth. In this example, to the left of the top strut, the endothelium has recovered quickly and proliferation has nearly stopped. On the right and at the bottom, proliferation is ongoing. Green cells are in the cell growth and proliferation cycle, with dark green cells having endothelium cover. Blue cells are endothelium-covered and have been inhibited by nitric oxide concentration as a result of high wall shear stress (see below). Orange cells are quiescent.

After each step of the cell model, a fixed lattice-based representation of the domain is constructed, with agent-covered nodes marked as solid. A constant parabolic velocity profile is set on the inlet, and a Lattice Boltzmann solver is used to compute the wall shear stress (WSS) at each SMC adjacent to the flow under the assumption that the total flux through the arterial segment remains constant as the restenosis develops. At locations where an intact and functional endothelium is present, nitric oxide (NO) is produced, which in turn inhibits SMC growth if its concentration is high enough.^31^ The drug diffusion submodel, which operates on the same lattice, simulates antiproliferative drugs eluting from the stent (if any) and diffusing through the tissue, inhibiting proliferation.^5^ SMC growth is also inhibited if the SMC is surrounded by other cells.

Following one of the approaches in experimental studies, we base our assessment of restenosis on the remaining cross-sectional lumen area.^24^ We consider a restenosis to have taken place if the final cross-sectional area of the neointima is more than 50% of the original cross-sectional lumen area of the vessel. To estimate this area at a given simulation time, we measure the mean width of the lumen at that time, and estimate the cross-sectional area of the lumen as that of a circle with that diameter. We then subtract this from the initial post-stenting lumen area, to obtain the area of the neointima.

### Model Set-up

Our simulation set-up is essentially the same as in the work of Tahir *et al*.^31^ We configured the model to represent a small section of a healthy porcine coronary artery, with a length of 1.5 mm, a lumen diameter of 1 mm, and a tunica media consisting of five layers of SMCs. A healthy porcine artery was selected to better facilitate the model’s validation, since the histological data points from the corresponding *in vivo* experiments are readily available. We used a bare metal stent with a mean deployment depth of 110 *µ*m. The blood flow model was set to dynamic viscosity *µ* = 4 mPa s, density $\rho$ = 1000 kg m^−3^ and Re = 120, resulting in a mean steady flow velocity of 0.48 m s^−1^.

**Figure 2:**
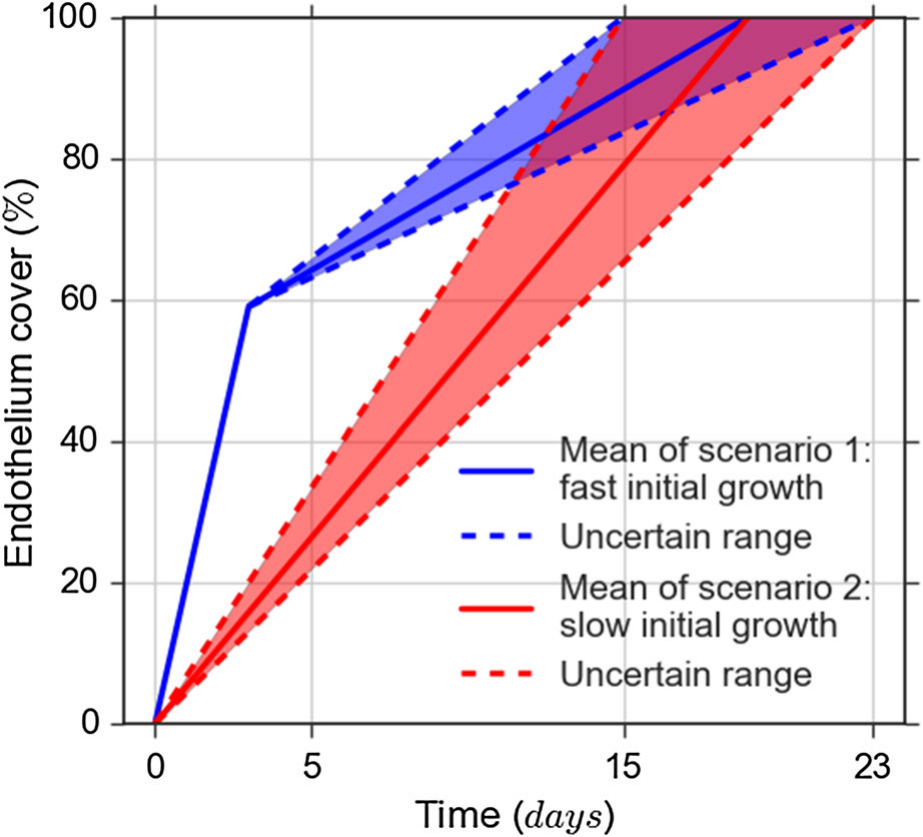
Two re-endothelialization scenarios, showing the percentual endothelium coverage over time. The uncertainty in the scenarios is also shown and is expressed by the uncertainty at which time 100% coverage is reached.

We considered two re-endothelialization scenarios (Fig. 2). In the first scenario, the endothelium recovers to a coverage of 59% after 3 days, followed by a full recovery after 15 to 23 days (determined by an uncertain input parameter, see Table 1). In the second scenario, the initial fast recovery is absent, with endothelium cover increasing linearly from zero to 100% after 15 to 23 days. These scenarios correspond to the three endothelium recovery cases of Tahir *et al*.^31^ The uncertainty range in Scenario 1 was chosen to cover the space between their cases 1 and 2.

In the model, reendothelialization is driven by three parameters: the initial fast recovery time, initial fast recovery degree, and the time to total recovery. Rather than having two scenarios, we could of course have taken the initial fast recovery time and degree as additional uncertain parameters, which would be appropriate as the measurement they are based on has a high relative error.^33^ However, reducing the number of uncertain parameters reduces computational cost, and with the present set-up, we can demonstrate a comparison between scenarios in the presence of uncertainty.

### Aleatory Uncertainty in the Model

The model is stochastic because the length of the cell cycle is chosen randomly for each SMC (normal distribution, $\mu = 32$ h, $\sigma = 2$ h), the relative orientation of daughter cells is chosen randomly during mitosis, and the pattern of reendothelialization is random as well. For a detailed description of the model, we refer to previous publications.^5^,^31^

### Epistemic Uncertainty in the Model

For our uncertainty quantification and sensitivity analysis of the ISR2D model, we consider the uncertainty in three input parameters (Table 1). Stent deployment depth and endothelium regeneration time were shown to strongly affect simulated neointimal area by Tahir *et al*.,^29^ and were therefore included. Additionally, we included blood flow velocity, because its effect on the behavior of the model has not yet been evaluated, and because of the potentially interesting interaction with the endothelium regeneration time. Blood flow in the coronaries is also variable depending on oxygen demand in the heart muscle, and by treating the blood flow as an uncertain parameter, we capture this effect into our analysis.

**Table 1:** List of uncertain inputs, where a uniform distribution is assumed between the minimum and maximum values.

Parameter	Unit	Min value	Max value	Reference
Flow velocity	m s^−1^	0.432	0.528	20
Maximum deployment depth	mm	0.09	0.13	9
Endothelium regeneration time	days	15	23	Fig. 2

Flow velocity was varied by 10%. The other ranges were chosen to correspond to previously published results,^31^ so that we could validate our findings.

## Uncertainty Quantification

Here we provide some relevant details of the Uncertainty Quantification methods we applied. We describe our uncertainty measures of the total model response uncertainty, estimation of aleatory and epistemic uncertainty in the model result, and sampling scheme. In this section, the ISR2D model, described in “The ISR Model”, is denoted by function $f(\xi , {\mathbf{x}})$, which depends on a vector of stochastic model parameters $\xi$ described in “Aleatory Uncertainty in the Model” and uncertain model inputs ${\mathbf{x}}$ from Table 1 (Section “Epistemic Uncertainty in the Model”).

### Uncertainty Measures

**Figure 3:**
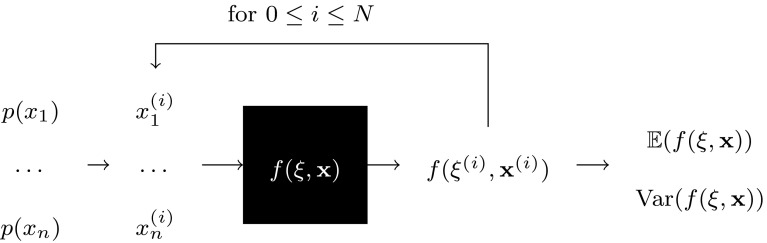
Black-box Monte Carlo uncertainty quantification flowchart: at each Monte Carlo run *i*, the uncertain inputs $x_j^{(i)}$ (for $1 \le j \le n$ with *n* number of uncertain inputs) take a random value according to their distribution $p(x_j)$. The model is run, and the stochastic parameters $\xi$ take some random values during the simulation. Then, the model produces a value of the output $f(\xi ^{(i)}, {\mathbf{x}}^{(i)})$. We run model in parallel *N* times. Using these *N* samples, we are able to estimate the uncertainty measures of the model result.

In this study, we used the variance (${\mathrm{Var}} (f(\xi , {\mathbf{x}}))$), standard deviation ($\sqrt{{\mathrm{Var}} (f(\xi , {\mathbf{x}}))}$), and the coefficient of variation (*CV*) as measures of model response uncertainty:

1 $$\begin{aligned} CV =\frac{\sqrt{{\mathrm{Var}}(f(\xi , {\mathbf{x}}))}}{|{\mathbb{E}}(f(\xi , {\mathbf{x}}))|} \cdot 100\%, \end{aligned}$$

where ${\mathbb{E}}(f(\xi , {\mathbf{x}}))$ is the mean value of the model response. These measures were estimated using a Monte Carlo method (Fig. 3) by running the model with different values of ${\mathbf{x}}$ and $\xi$, and collecting samples of the model results, in order to estimate the result uncertainty.

### Aleatory Uncertainty Estimation

*Aleatory* uncertainty is the type of uncertainty arising from natural variability of the model output.^19^ We collectively denote the stochastic variables that describe such variability in the model, as $\xi$. We can apply Saltelli’s method^22^ in order to measure the part of variance (${\mathrm{Var}}^T_{\xi }$), which would remain even if all uncertainty in the model inputs is removed:

2 $$\begin{aligned} {\mathrm{Var}}^T_{{\mathbf{\xi }}} \approx \frac{1}{2M} \sum _{j=1}^M{ \left( f({\mathbf{x}}^j ,\xi ^j) - f({\mathbf{x}}^j ,\xi ^{j+M})\right) ^2}, \end{aligned}$$

where $f({\mathbf{x}}^j ,\xi ^j)$ and $f({\mathbf{x}}^j ,\xi ^{j+M})$ are the model results with the uncertain inputs having the same values ${\mathbf{x}}^j$, but stochastic parameters having different values $\xi ^{j}$ and $\xi ^{j+M}$. *M* is the number of evaluations of the difference in 2.

Additionally, we are interested in the partial standard deviation ($\sigma ^T_{{\xi }}$), since it has the same units as the mean value. In general, we can approximate this value using a brute force Monte Carlo approach, but this is computationally expensive. However, Jensen’s inequality^11^ provides us a way to control from above the partial standard deviation with the square root of the partial variance ${\mathrm{Var}}^T_{{\xi }}$:

3 $$\begin{aligned} \sigma ^T_{{\xi }}= & {} E_{\mathbf{x}}\left( \sqrt{{\mathrm{Var}}_{\xi }(f(\xi , {\mathbf{x}})|{\mathbf{x}})}\right) \nonumber \\\le & {} \sqrt{E_{\mathbf{x}}\left( {\mathrm{Var}}_{\xi }(f(\xi , \mathbf {x})|{\mathbf{x}})\right) } = \sqrt{{\mathrm{Var}}^T_{{\xi }}}. \end{aligned}$$

### Epistemic Uncertainty Estimation

*Epistemic* uncertainty is model imprecision, which arises due to lack of knowledge.^19^ In this study, uncertain values of model inputs ${\mathbf{x}}$ generate such epistemic uncertainty. In order to estimate the parts of uncertainty arising due to imprecision in the value of each of the model inputs, we applied the Sobol variance-based sensitivity analysis method.^26^ The Sobol Sensitivity Indices (SIs) are the ratio between the partial variance and the variance of the model function $f(\xi , {\mathbf{x}})$. In this work, we compute the first-order ($S_{x_i}$) and total ($S^T_{\xi , x_i}$) SI for each of the uncertain model inputs, which are

4 $$\begin{aligned} S_{x_i}=& {} \frac{{\mathrm{Var}}_{x_i}}{{\mathrm{Var}}(f(\xi , {\mathbf{x}}))} \cdot 100 \%,\nonumber \\ S^T_{\xi , x_i}=& {} \frac{{\mathrm{Var}}^T_{\xi , x_i}}{{\mathrm{Var}}(f(\xi , {\mathbf{x}}))} \cdot 100 \%, {\text{for }} i = 1 \cdots n, \end{aligned}$$

where ${\mathrm{Var}}_{x_i}$ and ${\mathrm{Var}}^T_{\xi , x_i}$ are the partial variances, and *n* is the number of uncertain inputs. These partial variances for a parameter $x_i$ can be expressed by Refs. 22 and 23

$$\begin{aligned} {\mathrm{Var}}_{x_i} = {\mathrm{Var}}_{x_i}(E_{\xi , {\mathbf{x}}_{-i} }(f(\xi , {\mathbf{x}})|x_i)), \end{aligned}$$

which is the expected reduction in variance, if $x_i$ were known precisely, and

$$\begin{aligned} {\mathrm{Var}}^T_{\xi , x_i} = E_{{\mathbf{x}}_{-i}}({\mathrm{Var}}_{\xi , x_i}(f(\xi , {\mathbf{x}})|\mathbf {x}_{-i})), \end{aligned}$$

which is the expected remaining variance, if all the parameters except $x_i$ were known exactly.^1^${\mathbf{x}}_{-i}$ denotes a vector of all inputs except $x_i$.

We can approximate the partial variances for the first-order and total SI for the *i*th parameter by Ref. 27

5 $$\begin{aligned} {\mathrm{Var}}_{x_i}\approx & {} \frac{1}{M} \sum _{j=1}^M \left( f(\xi ^{j}, {\mathbf{x}}^j)-f_0 \right) \left( f(\xi ^{j+M}, {\mathbf{x}}_{-i}^{j+M}, x^{j}_i)-f_0 \right) , \nonumber \\&\text { with } f_0 = \frac{1}{M} \sum _{k=1}^M {f(\xi ^{k}, {\mathbf{x}}^k)},\end{aligned}$$

6 $$\begin{aligned} {\mathrm{Var}}^T_{\xi , x_i}\approx & {} \frac{1}{2M} \sum _{j=1}^M {\left( f(\xi ^{j}, {\mathbf{x}}^j) - f(\xi ^{j+M}, {\mathbf{x}}_{-i}^j, x^{j+M}_i)\right) ^2}, \end{aligned}$$

where $f(\xi ^{j+M}, {\mathbf{x}}_{-i}^{j+M}, x^{j}_i)$ is the result with input $x_i$ having the same value as for $f(\xi ^{j}, {\mathbf{x}}^j)$, but the rest of the model parameters having different values. $f(\xi ^{j+M}, {\mathbf{x}}_{-i}^j, x^{j+M}_i)$ denotes the model outputs with the same values of all inputs as for $f(\xi ^{j}, {\mathbf{x}}^j)$, except for the values of $\xi$ and $x_i$.

The total number of the model evaluations required for computing both aleatory and epistemic uncertainties is $M (2n + 2)$, which is equal to *N* in this work.

### Sampling

Instead of uniform random numbers, we used the Sobol sequence.^25^ This sequence is called *quasirandom*, since it is not random, but preserves enough properties of random numbers to be used in Monte Carlo methods. The Sobol sequence is *low-discrepancy*, which means that it covers input space $\Omega$ more evenly.

For quasirandom sequences, convergence is of the order ${\mathscr{O}}\left(\frac{1}{N}\right)$, where *N* is the number of samples, compared to ${\mathscr{O}} \left(\frac{1}{\sqrt{N}}\right)$ for random numbers,^15^ resulting in a lower approximation error for a given number of samples. However, the confidence interval of the estimators^2^ can no longer be approximated by the ratio $\frac{\sigma }{\sqrt{N}}$.

Instead, we performed a bootstrap test.^1^ Originally we have *N* samples of the model results. We randomly select *N* samples from this original set with replacement. The estimator is computed using this new collection of model results, and we call $I_k^{*}$ the *k*th approximation of an estimator. After obtaining *K* re-evaluations of the estimator, we compute the estimators’ mean ($\bar{I}$) and standard deviation ($\varepsilon(\bar{I})$):

7 $$\begin{aligned} \bar{I}= & {} \frac{1}{K}\sum ^K_{k=1}{I_k^{*}},\nonumber \\ \varepsilon (\bar{I})= & {} \sqrt{\frac{1}{K-1} \sum ^K_{k=1} {(I_k^{*}-\bar{I})^2}}. \end{aligned}$$

Thus, the precision of the estimator obtained with *N* samples is

8 $$\begin{aligned} \bar{I} \pm \varepsilon (\bar{I}). \end{aligned}$$

Reliable values of $\bar{I}$ and $\varepsilon (\bar{I})$ can be obtained with the bootstrap when *K* is a large number. We chose *K* equal to 10,000.

In our experiment, we first set the sample size *N* to 480. The bootstrap test of the confidence in the mean value and standard deviation estimators showed that the estimators’ standard deviations ($\varepsilon (\bar{I})$) were up to 10%. We doubled the number of samples to $N = 960$, resulting in standard deviations not exceeding 2.3%. This is deemed precise enough, and all results are based on using 960 samples.

We ran the ISR2D model once for each sample, for a total of 960 runs. The model is configured *via* a configuration file that contains a description of the model structure as well as values for its input parameters. We used a custom Python script to generate these configuration files, using the SOBOL library^21^ to generate the Sobol quasirandom sequences.^28^ As the runs are independent, they were run in parallel, using a single core for each run for maximum efficiency. Runs took on average 2 h and 15 min to complete, for a total of  4300 core hours. Approximately 100 more core hours were spent on postprocessing the results.

## Results

We observe a single output parameter of the model, the cross-sectional area of the neointimal growth, as a function of time. We compare the two endothelial recovery scenarios, taking into account the uncertainty of the model output, and estimate the contributions of the aleatory and epistemic uncertainty. We then investigate the relative importance of the input parameters as well as the inherent model stochasticity, in a sensitivity analysis. Finally, we show the spatial distribution of (explained) uncertainty.

### Neointimal Growth

**Figure 4:**
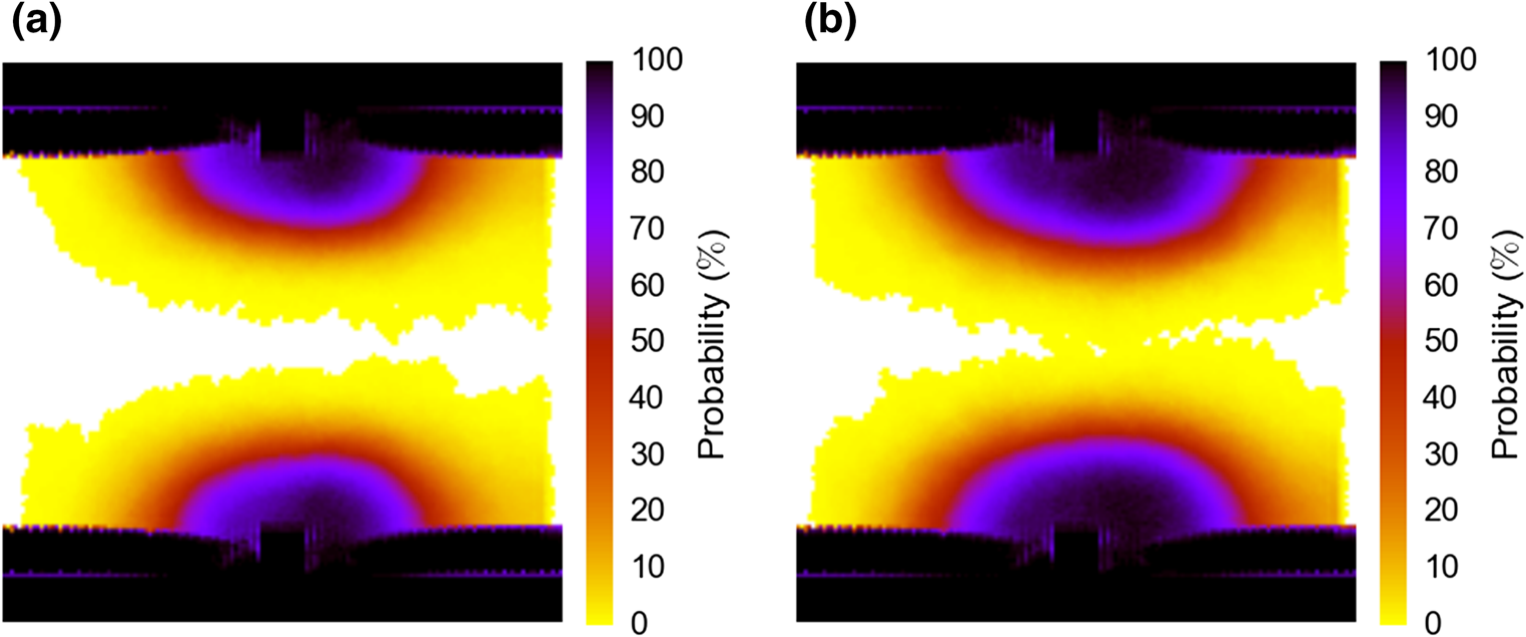
Probability of presence of SMCs in the simulated domain at the final simulation time step for respectively re-endothelialization Scenario 1 (left) and Scenario 2 (right).

Figure 4 shows the probability of SMC presence over the domain at the end of the simulation run, giving an idea of the shape and size of the final neointimal growth produced by the model. Colors show the fraction of samples in which the given location was covered by neointima. On the whole, the results are similar for the two scenarios, an oval-shaped neointima surrounding the stent strut, shifted somewhat in the direction of the blood flow. As expected, the slower endothelium recovery in Scenario 2 leads to larger overall growth. Since this is a longitudinal view, the area difference is distorted; see below for a quantitative analysis. Note that in Scenario 2, it appears that the vessel can become fully closed. In fact, the top and bottom halves of the neointima sometimes form asymmetrically, and these different asymmetric runs are responsible for the innermost portions of neointima. In reality, the wall shear stress-induced growth inhibition will keep the lumen from closing up entirely in individual instances of the model.

### Uncertainty Propagation

**Figure 5:**
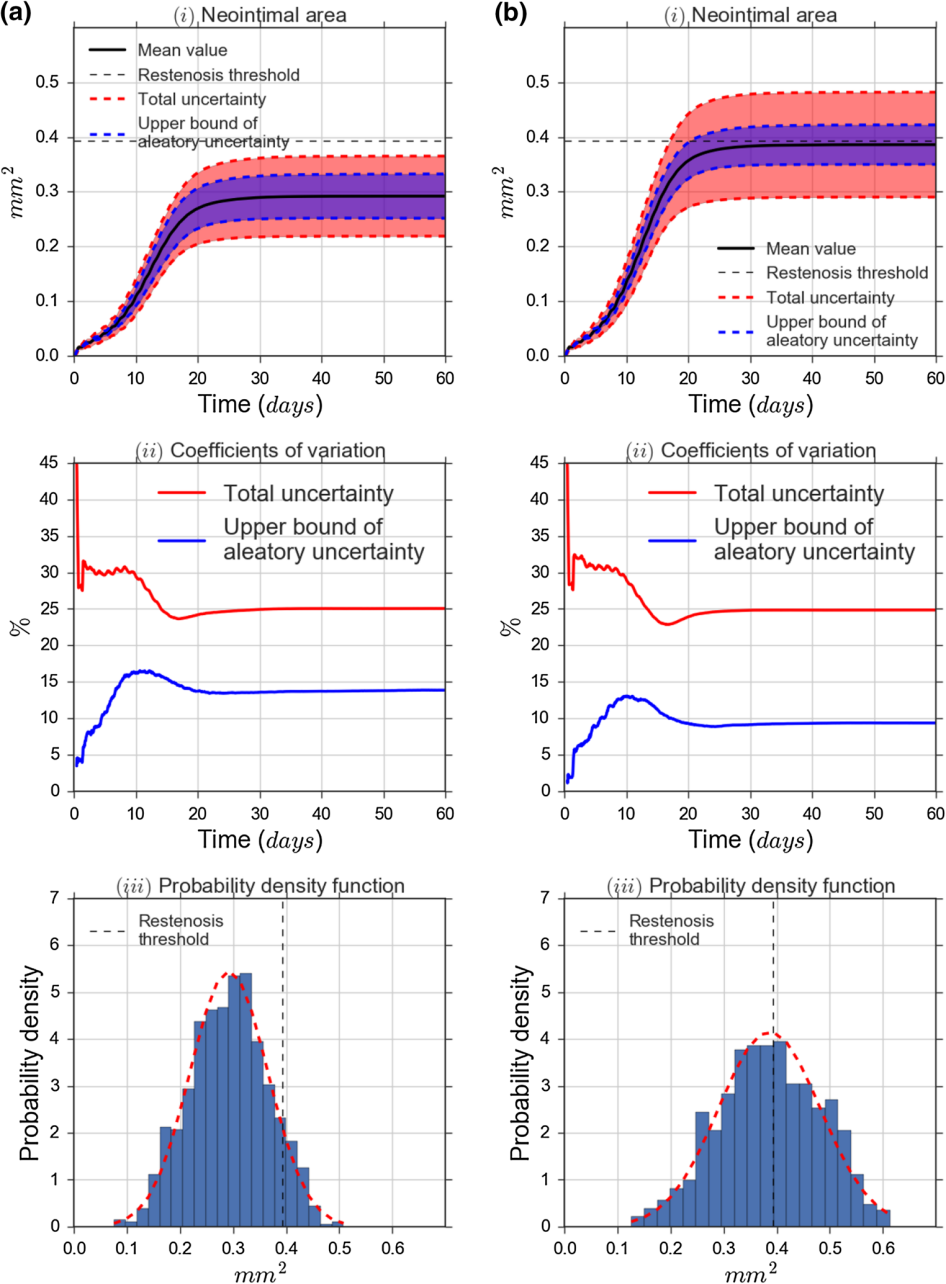
Uncertainty estimation results: (*i*) mean value, aleatory and total uncertainty of the neointimal area as a function of time; (*ii*) relative total uncertainty (red line) and relative aleatory uncertainty (blue line) as a function of time; (*iii*) Probability Density Function (PDF) of the neointimal area at the final time step, where red dashed curve is a fit of a normal distribution.

Figures 5(i) show the neointimal area over time as well as its estimated uncertainty. Initially, growth is slow as only a few SMCs are active, but as the size of the neointima increases, so does its growth rate (black line). Growth slows down again as the endothelium recovers and SMCs are increasingly inhibited, until a final state is reached. As expected, Scenario 2 leads to higher growth than Scenario 1 ($\approx$ 0.38 mm^2^ and $\approx$ 0.28 mm^2^ at the final time step). For the 1 mm vessel we simulated, a 50% cross-sectional area reduction corresponds to a neointimal area of approximately 0.39 mm^2^ (horizontal dashed line). For both scenarios, the mean neointimal area is below this restenosis threshold.

Overall uncertainty is fairly high however, with a standard deviation of 0.07 mm^2^ for Scenario 1, and 0.09 mm^2^ for Scenario 2 (red dashed lines). There are two sources of this uncertainty: the three uncertain input parameters give rise to epistemic uncertainty, while the internal stochastic variables result in aleatory uncertainty. Our results show that if the uncertain parameters were known exactly, the remaining aleatory standard deviation in the final state would be at most $\approx$ 0.04 mm^2^ in both scenarios (purple dashed lines). With respect to the occurrence of restenosis, we can conclude that in Scenario 1 a restenosis occurs in less than 10% of cases, while in Scenario 2 the probability is close to 50%.

The relative uncertainty (coefficient of variation, Eq. (1)) is depicted in Fig. 5(ii). Total relative uncertainty (red lines) is initially around 30%, then drops to $\approx$ 25% as the growth slows and the slower growing runs catch up to some extent. The serrations on the left are due to all SMCs starting their cell cycle simultaneously; they later smooth out due to the variability in cycle length.

As stent deployment is only affected by deployment depth and not by any internal stochasticity, relative aleatory uncertainty (blue lines) starts out at zero. The aleatory uncertainty increases as SMCs start to proliferate and the stochastic variables come into effect, then drops slightly as the total uncertainty does to settle at $\approx$ 14% and $\approx$ 9% respectively. The final absolute aleatory uncertainty is actually nearly the same for both scenarios, but as the total area is larger in Scenario 2, the relative uncertainty is lower.

The probability density functions for the final neointimal area (Fig. 5(iii)) show nearly normal shape, validating our choice to use the variance as a measure of uncertainty.

### Sensitivity Analysis

**Figure 6:**
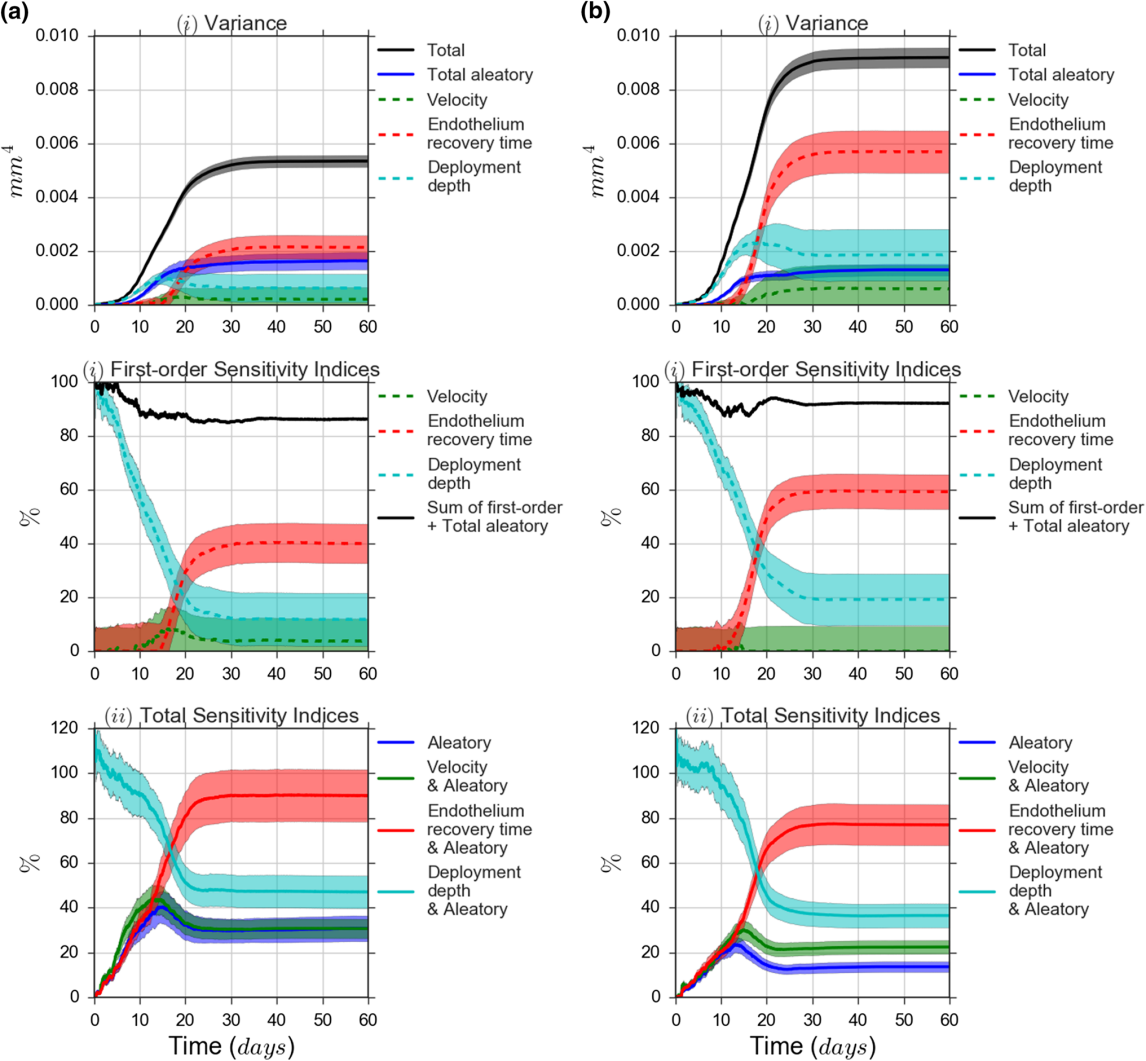
Sensitivity analysis with the Bootstrap test results: (*i*) partial and total variances; (*ii*) first-order sensitivity indices of uncertain inputs, where solid black line denotes the sum of the first order effect of uncertain inputs plus the total effect of the model stochastic variables; (*iii*) total sensitivity indices of uncertain parameters together with the stochastic variables. The shadow areas is one standard deviation of the estimators.

The sensitivity analysis results over simulation time are shown in Fig. 6. For each of the uncertain inputs, we computed the first order Sensitivity Index $S_{x_i}$, as well as the total sensitivity index $S^T_{\xi , x_i}$, the latter including the combined stochastic model variables $\xi$. Shaded bands indicate a one standard deviation uncertainty interval of the estimate. Although at the number of samples we took there is a fairly large amount of uncertainty in the estimates, the relative importance of the input parameters is clear.

Figure 6(i) show the variance in the neointimal area as a function of time. As we saw before, the total variance (black) is larger for Scenario 2 than for Scenario 1. The total (absolute) aleatory uncertainty (blue) is the same however. This measure includes the aleatory uncertainty by itself, and all combined effects of the aleatory uncertainty and the uncertain inputs. If all uncertain inputs were known exactly, this would be the remaining variance of the neointimal area.

For the final state of the simulation, the endothelium recovery time (red) is the most important parameter in both scenarios, followed by stent deployment depth (cyan) and flow velocity (green). This order is different in the beginning of the simulation, but this is difficult to see as the overall variance is close to zero.

Figure 6(ii) show the first order sensitivity indices of the input parameters. As these are relative to the total variance, the relative importance over time can be studied more easily, although there is some noise for the early stages. Still, for both scenarios, it is now clear that the deployment depth explains almost all variance at the start of the growth process, while regeneration time becomes more important during the later stages. This makes sense, as the deployment depth affects the initial activation of the SMCs, while the regeneration time influences their inhibition. The importance of the flow velocity remains low throughout, which would suggest a low sensitivity to physiological variability of the blood flow. The importance of the regeneration time relative to the other parameters appears to be larger for Scenario 2; this is mostly due to the smaller relative share of the aleatory uncertainty however.

The black line in these graphs is the sum of the three first-order sensitivity indices, plus the total aleatory uncertainty. It thus represents all the sources of uncertainty except for the higher order combined effects of the input parameters. This shows that these combined effects explain at most in the order of 20% of the uncertainty.

In Fig. 6(iii), the total sensitivity indices (TSIs) for the input parameters are shown, which include all first-order and combined effects, including combined effects with the stochastic variables. Here we see the same pattern, with deployment depth dominating in the early stages, and regeneration time in the late stages of the simulation. A bump in the aleatory TSI is visible at days 10–15, and since this is included in the other plotted values, shows up in them as well. This is likely due to the stochastic variation in the SMC cell cycle lengths, which results in some simulation runs growing faster than others. Near the end of the growth phase, the slower runs catch up, and the variance decreases again. For Scenario 2, the flow velocity now shows a clear effect. This must be a combined effect, presumably with the regeneration time, as the timing is correct and the mechanism clear. For Scenario 1, flow velocity only shows a small combined effect at around day 7.

### Spatial Distribution of Uncertainty

**Figure 7:**
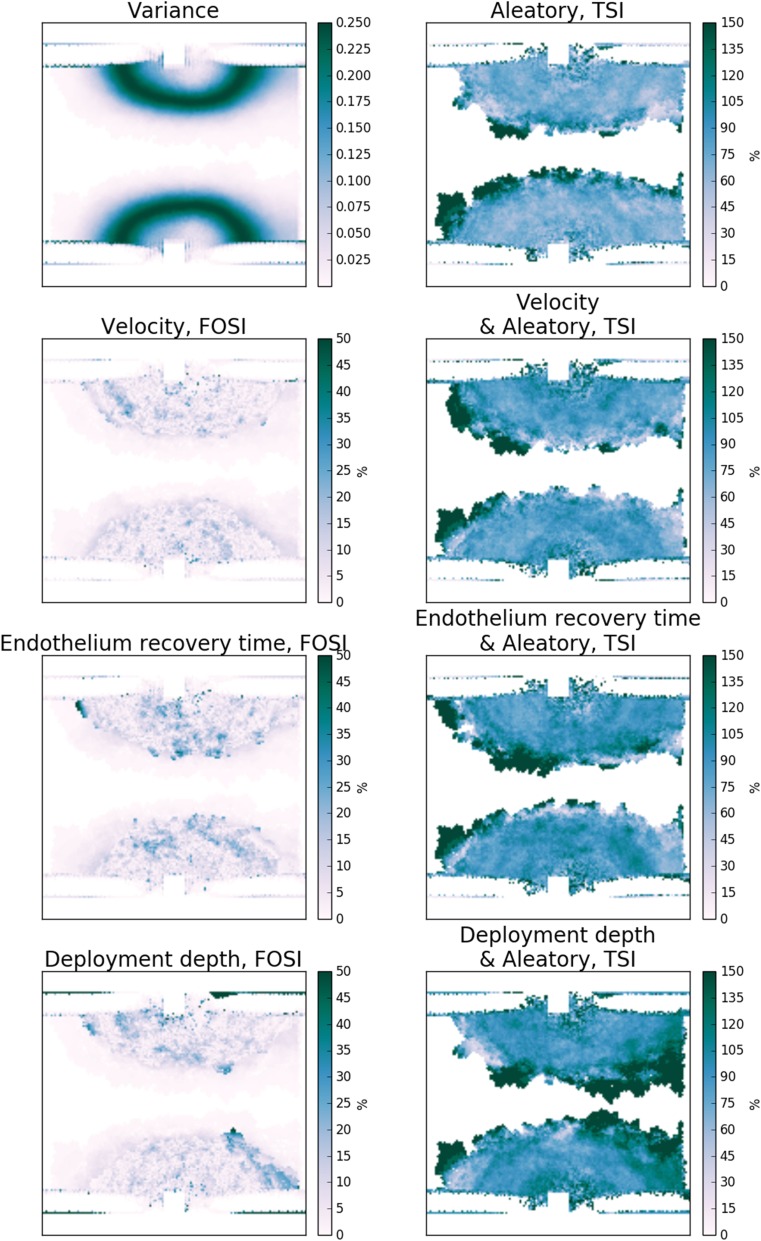
Sensitivity maps for Scenario 1 at the final time step: variance, first-order (FOSI) and total (TSI) sensitivity indices of the neointimal area for each of the uncertain model parameters.

**Figure 8:**
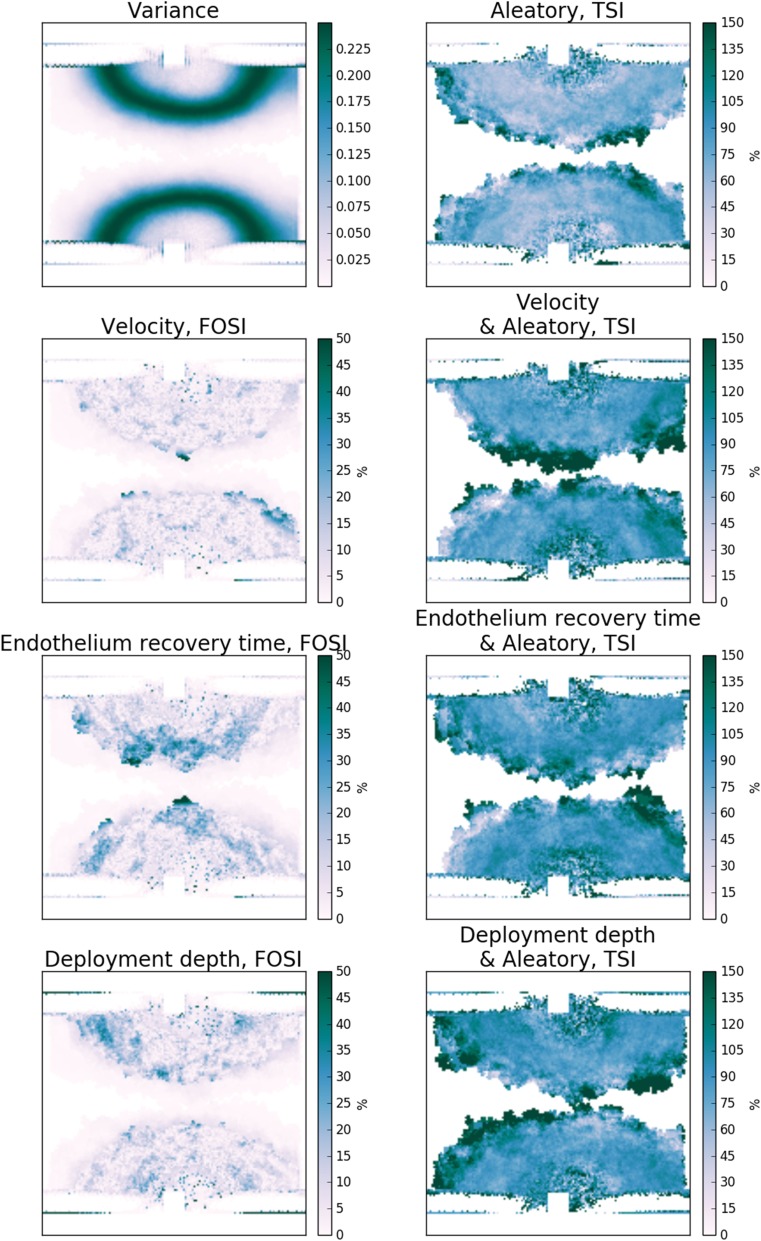
Sensitivity maps for Scenario 2 at the final time step: variance, first-order (FOSI) and total (TSI) sensitivity indices of the neointimal area for each of the uncertain model parameters.

Figures 7 and 8 show the variance and SI in space for the final time step of the simulation for Scenarios 1 and 2, respectively. We can conclude that none of the uncertain inputs bring high uncertainty in a particular area of the observed space solely. In the results for the first scenario, we observe that the total SI for the deployment depth shows especially high value on the outflow side of the SMC growth. Moreover, we see that the SI of the deployment depth in average over space shows a higher value in comparison of the SI for other parameters.

## Discussion and Conclusions

We performed uncertainty quantification and sensitivity analysis for a 2D in-stent restenosis model, demonstrating how the variation of a few selected parameters of the model affects the simulation results as well as whether knowing the exact value of a parameter is important for the precision of the simulation results.

From the sensitivity analysis we find that the largest contribution to the uncertainty comes from the endothelium regeneration speed, followed by the deployment depth. This is similar to results obtained in an earlier studies for a similar 3D model.^34^ There it was also demonstrated that a difference in re-endothelialization speed changes growth to a large extent, while the effects of the deployment depth are smaller, but still quite pronounced. As the re-endothelialization has such significant impact on the neointimal area, this part of the ISR model requires further study and validation. We are currently undertaking more detailed modelling, based on controlled experiments of endothelial cell migration on substrates. The results once more confirm our hypothesis^31^ that development of a restenosis is very much driven by the inability to quickly regenerate a functional endothelium.

However, these results cannot be considered final. The model parameters were based on a previous publication,^31^ which considered a largely simplified model geometry, and some other parameters also were assumed without a thorough investigation on their distribution or variability range. For instance, the effect of the flow velocity requires a deeper study, looking in more detail at the actual physiological variability that may be expected (instead of the 10% variability that we now assumed). Additionally, it is hard to determine physiological values and draw conclusions about real systems for the 2D model, since the model considers a very simplified short and straight segment of the vessel. In real arteries, the curvature of the artery also plays a big role, causing uneven flow and also causing side effects such as hinging during the stent deployment.^12^ Also, the model calculates a steady flow in the vessel, while in reality pulsatile flow results in a time-varying WSS and sometimes even WSS reversal.^32^

Still, the results do show that once we have a reasonable understanding of the model and its parameters, we can extract important observations from the model (i.e. would a certain stent deployment lead to an actual restenosis) with a well defined uncertainty, even though this is a relatively involved multiscale model showing a complex biomechanical response. This method for uncertainty quantification and sensitivity analysis will therefore contribute strongly to the validation of these kinds of models.

On the other hand, we need to acknowledge the limited validity of the 2D model we used. We should therefore apply the methods described in this paper to the 3D version of the ISR model,^34^ which is closer to the real system and also better validated. However, the 3D model contains millions of cell agents compared to a few thousand agents in the 2D model, and the 3D flow calculations are much more expensive as well. This makes the computational cost of Uncertainty Quantification and Sensitivity Analysis for the 3D model much higher or even prohibitive. We have therefore started to develop UQ algorithms for multiscale models that are capable of reliable estimation of the uncertainties while reducing the computational costs.^17^ We are planning to test these algorithms with the ISR3D model.

We conclude that quasi-Monte Carlo UQ and Sobol sensitivity analysis are reliable methods for estimating uncertainties in the response of complicated multiscale cardiovascular models such as the ISR. They can be used to determine which parameters affect the model results the most, and are therefore the most important ones to obtain precise measurements on. Given measurements of the parameters, output uncertainty can then be assessed. We believe that using these methods to assess the quality and usability of a simulation model is a crucial step on the way to certification and use in for instance in-silico clinical trials for coronary stenting.

## Footnotes

1 Note that the total sensitivity indices includes the contributions from the stochastic variable, since aleatory uncertainty is irreducible, and remains when values of other inputs are known precisely.

2 Here, the mean value, variances and standard deviation. For the sensitivity indices and partial variances, instead of sample size *N*, we test the number *M*, which is equal to the number of estimations of the expressions inside the sums in Eqs. (2), (5) and (6).
